# Supplementary material for: A cardiac transcriptional enhancer is repurposed during regeneration to activate an anti-proliferative program
Source: Development. 2025 Feb 17;152(4):DEV204458. doi: 10.1242/dev.204458 (PMC11883283; doi:10.1242/dev.204458)
Supplement: Supplementary information [file develop-152-204458-s1.pdf]

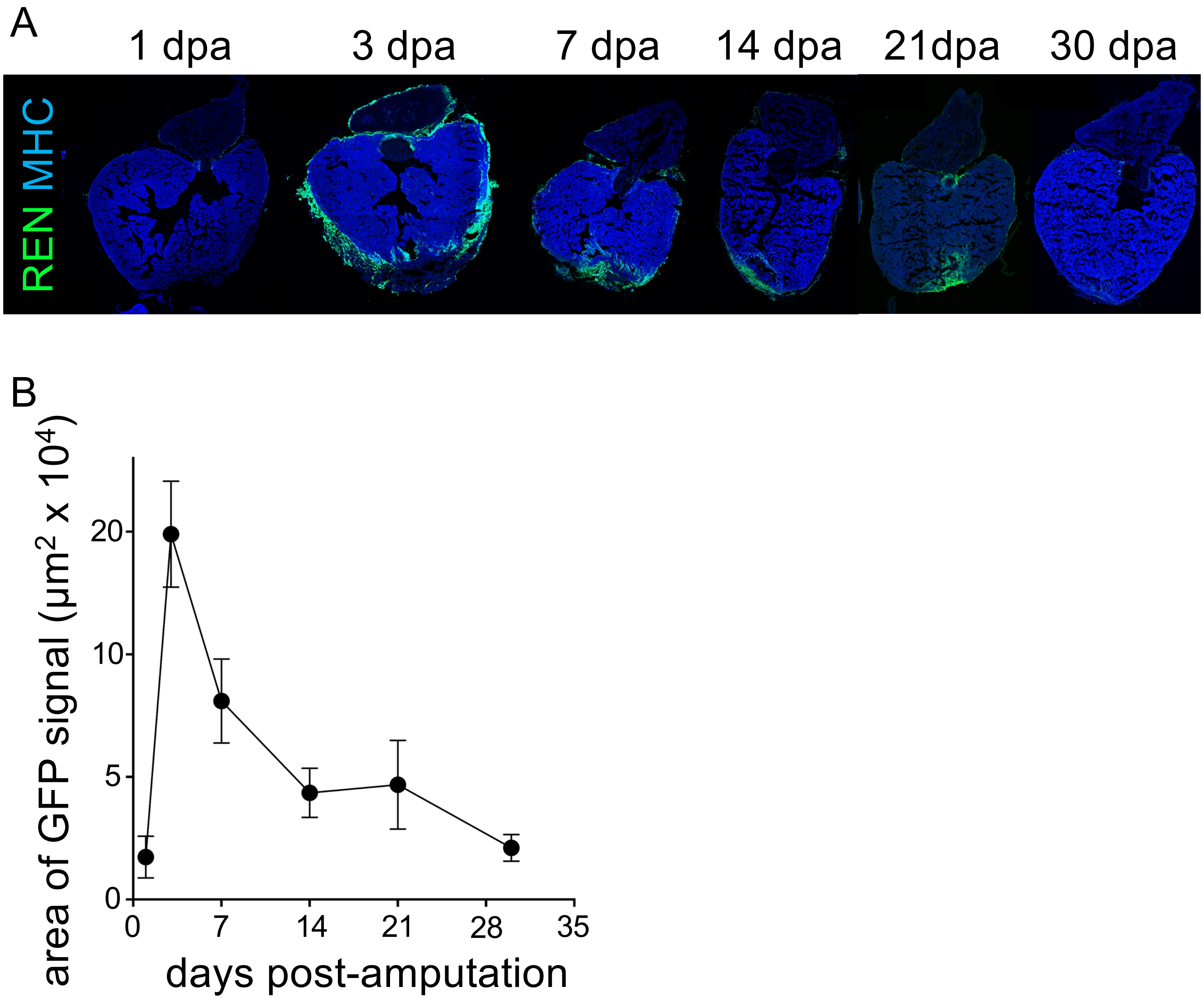

Fig. S1.
(A) Time course of *REN:GFP* expression throughout the heart during regeneration. REN – green, MHC – blue. (B) Quantification of total area containing GFP fluorescence.

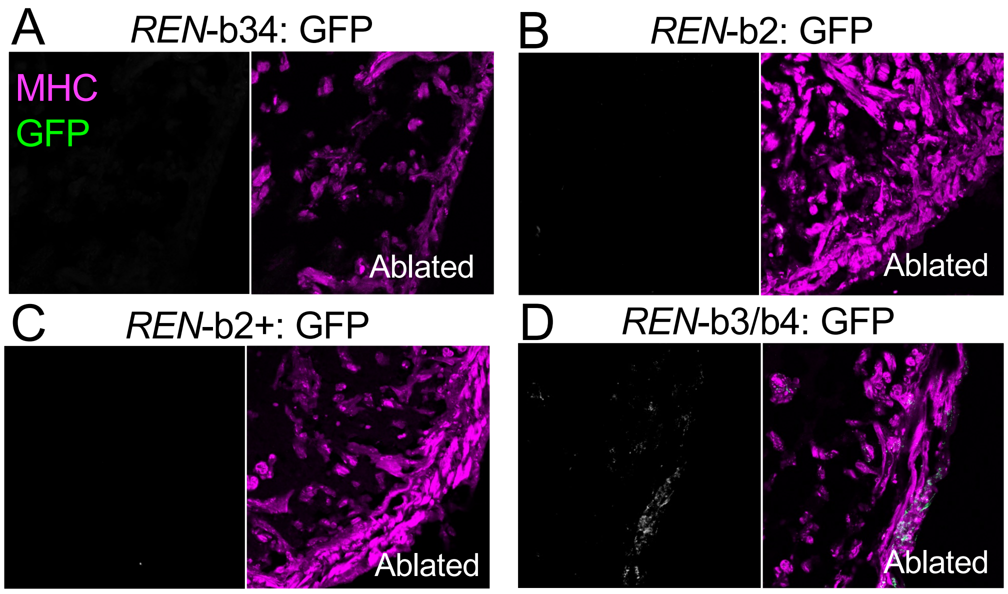

Fig. S2.

(A-D) Heart sections from the different REN fragments (labeled) in ZCAT hearts ablated 7 days-post-induction. Left – gray scale of GFP. Right – MHC (red), REN:GFP (green).

Development • Supplementary information

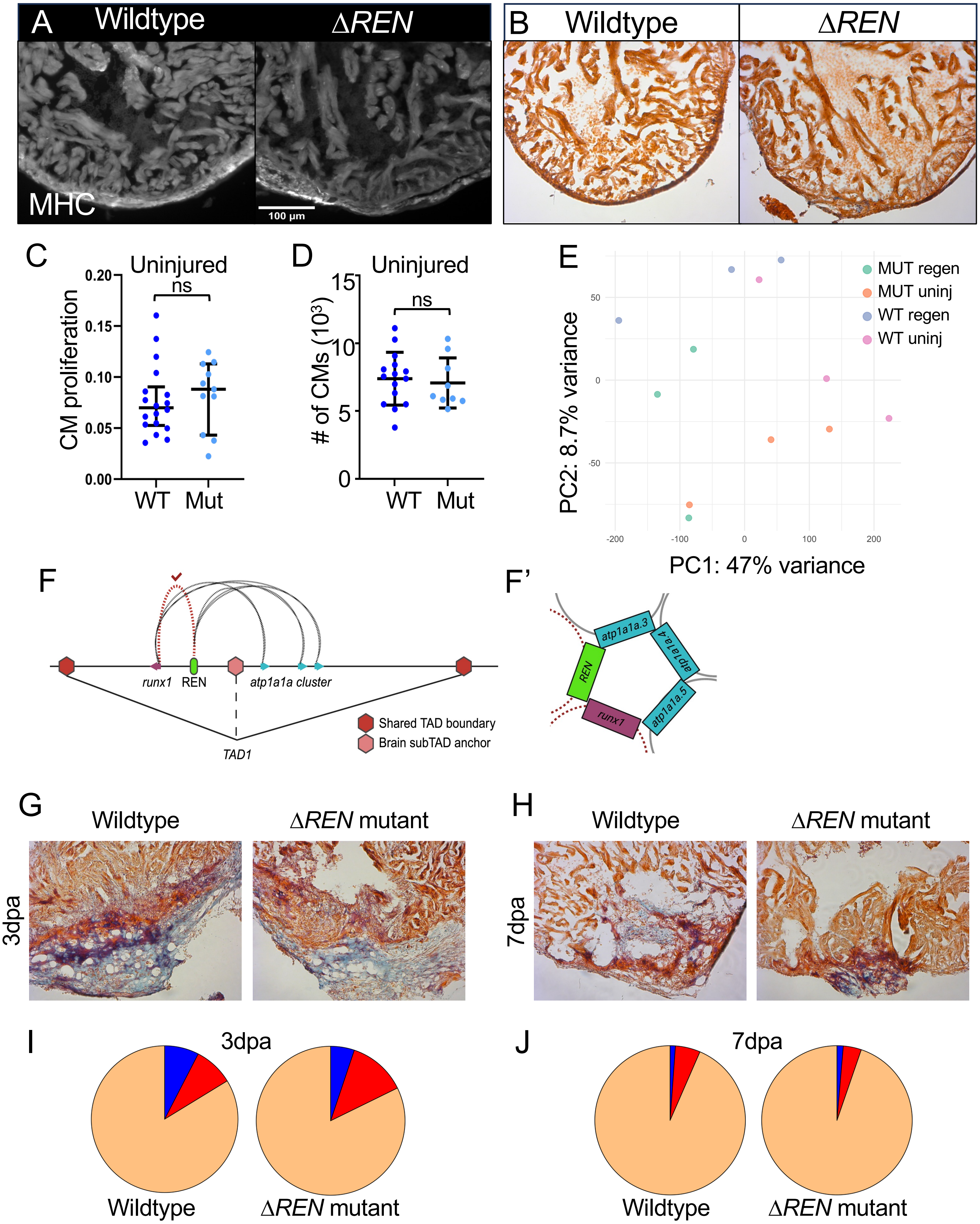

A Wildtype ΔREN
MHC
100 µm
B Wildtype ΔREN
C Uninjured
ns
CM proliferation
0.20
0.15
0.10
0.05
0.00
WT Mut
D Uninjured
ns
# of CMs (10^3)
15
10
5
0
WT Mut
E
MUT regen
MUT uninj
WT regen
WT uninj
PC2: 8.7% variance
PC1: 47% variance
F
runx1
REN
atp1a1a cluster
TAD1
Shared TAD boundary
Brain subTAD anchor
F'
atp1a1a.3
atp1a1a.4
atp1a1a.5
REN
runx1
G Wildtype ΔREN mutant
3dpa
H Wildtype ΔREN mutant
7dpa
I 3dpa
Wildtype ΔREN mutant
J 7dpa
Wildtype ΔREN mutant

Fig. S3.

(A) Wildtype and mutant hearts were stained with MHC 30 days-after-amputation. (B) AFOG staining of the same hearts. (C) Quantification of CM proliferation indices (Mef2/EdU double positive over total Mef2 positive) in uninjured ventricles (wildtype average = 0.78%; mutant average = 0.54%; Welch's t-test, p-value = .252, N = 7 vs 11). Wildtype – blue, mutant – light blue. Horizontal black bars display the mean (middle) or standard error (top and bottom). (D) Graph of total Mef2-positive CM numbers counts in adult uninjured hearts (average = 2095 and 1666; Welch's t-test, p-value = 0.115, N = 7 vs 11). (E) PCA analysis of RNAseq replicates. (F) Cartoon of the TAD detected at the end of chromosome 1 in brain and muscle that contains both *REN* and *runx1*. Genes whose promoters are found to interact with *runx1* are also included and interactions are indicated by the dashed lines. *REN* was also found to interact with *runx1* (and the other genes) in the same data sets from Yang et al. (F') Cartoon showing the '*REN* enhancer hub' where promoters for *runx1* and the three *atp1a1a* genes all interact with *REN* and each other. Created in BioRender by Goldman, A. (2025) (https://BioRender.com/z55t285) based on the data from Yang et al. This figure was sublicensed under CC-BY 4.0 terms. (G-H) Representative image of injury site from wildtype and Δ*REN* mutant hearts stained with AFOG at 3 days-post-amputation (G) and 7dpa (H). (I) Calculation of relative fibrin (red) and collagen (blue) levels from 3dpa based on Koth et al. methodology. wildtype averages: collagen = 7.75%; fibrin = 8.70%; muscle (orange) = 83.73%; mutant averages: collagen = 5.16%; fibrin = 12.60%; muscle (orange) = 82.25%; Chi-square p-value = 0.3376; N=7,5. (J) Calculation of relative fibrin (red) and collagen (blue) levels from 7dpa based on Koth et al. methodology. wildtype averages: collagen = 1.13%; fibrin = 5.36%; muscle (orange) = 93.51%; mutant averages: collagen = 1.35%; fibrin = 3.89%; muscle (orange) = 93.51%; Chi-square p-value = 0.825; N=5,6.

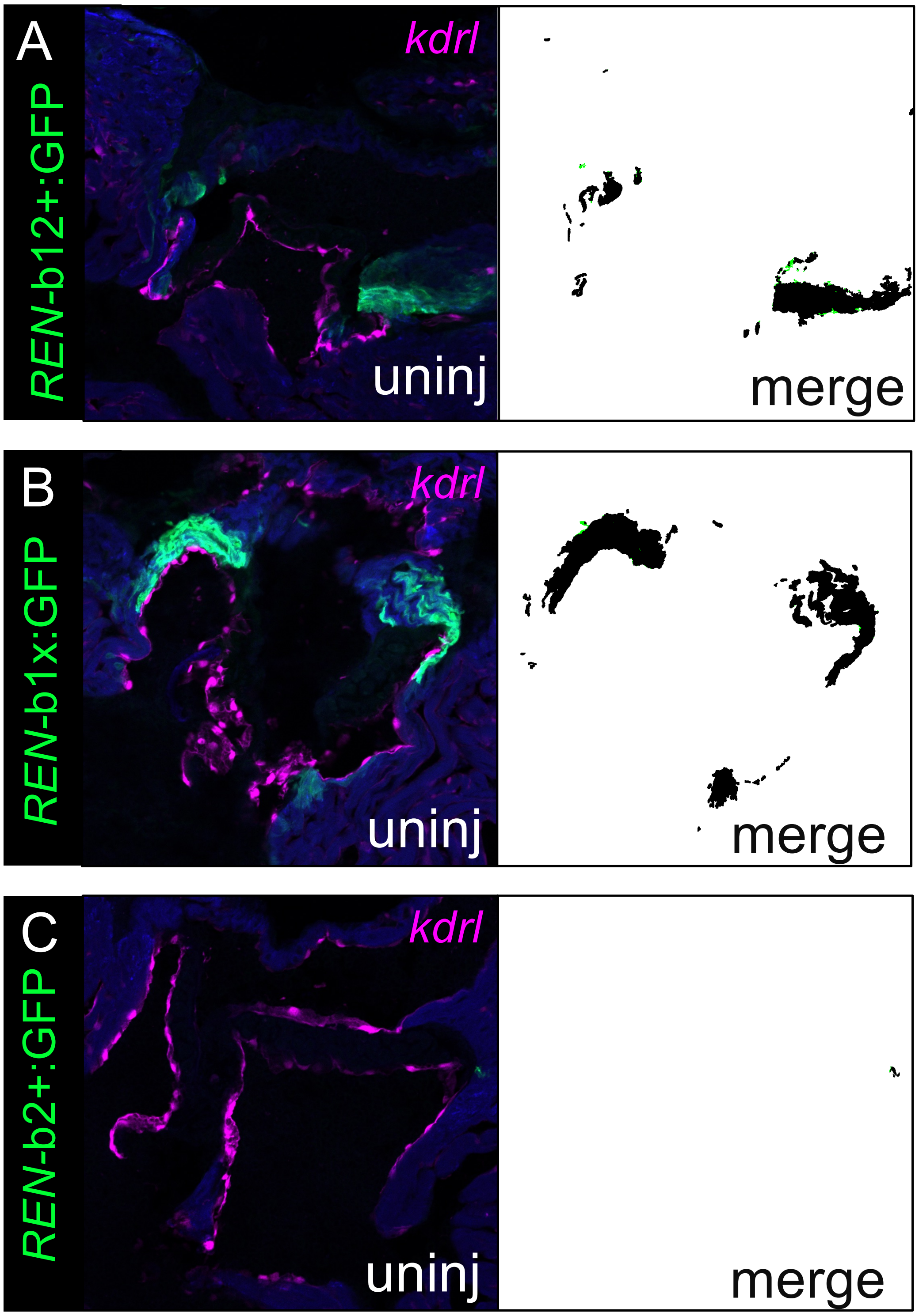

Fig. S4.
(A) Minimal fragment of REN is sufficient for CM expression around uninjured valves (REN-b1X); muscle (blue) and GFP (green). Right - MIPAR rendition of colocalized areas from REN-b1X are shown in black with excess GFP remaining in green. (B) REN fragments b12+ (C) and b2+.

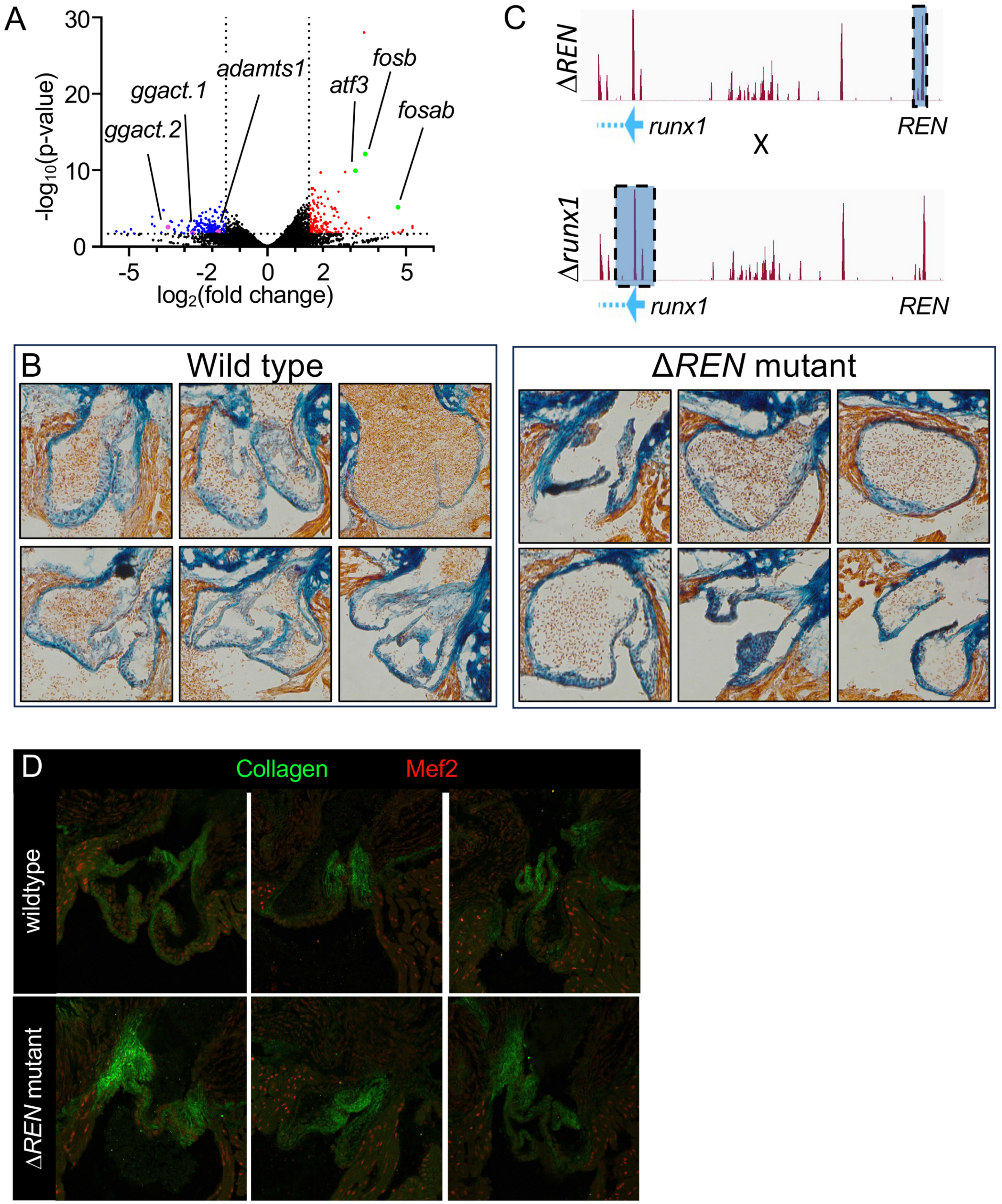

Development • Supplementary information

Fig. S5.

(A) Volcano plot showing differences in RNAseq from uninjured wildtype hearts vs uninjured Δ*REN* hearts. Transcripts of genes decreasing within 1.7Mb of REN on chromosome 1 are highlighted in pink and labeled with arrows. Members of the AP1 transcription factor complex are highlighted in green and labeled with arrows. (B) AFOG staining of uninjured wildtype and uninjured Δ*REN* mutant hearts. Images are zoomed in on the region around valves near the outflow tract. All replicates are included here to show support Figure 5DE. (C) Cartoon of complementation experiment. Shown are the regions of chromosome 1 deleted in the Δ*REN* and Δ*runx1* mutant lines (dashed blue boxes). The Y-axis is the enrichment of cardiomyocyte specific–histone H3.3 and the X-axis are coordinates along chromosome 1. (D) Immunofluorescence of cardiac valves with Collagen I (green) and Mef2c (red) antibodies. replicates are included here to support Figure 5F.

Development • Supplementary information

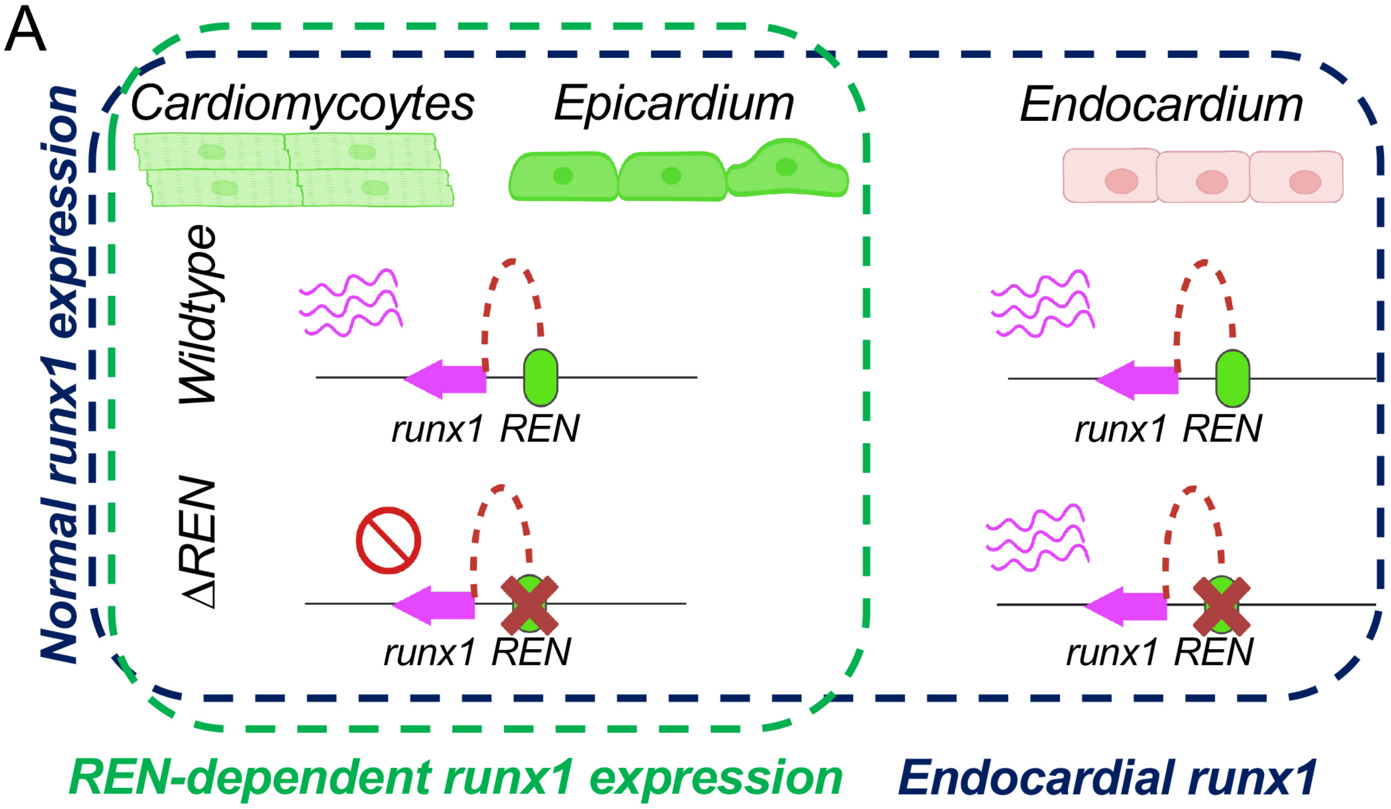

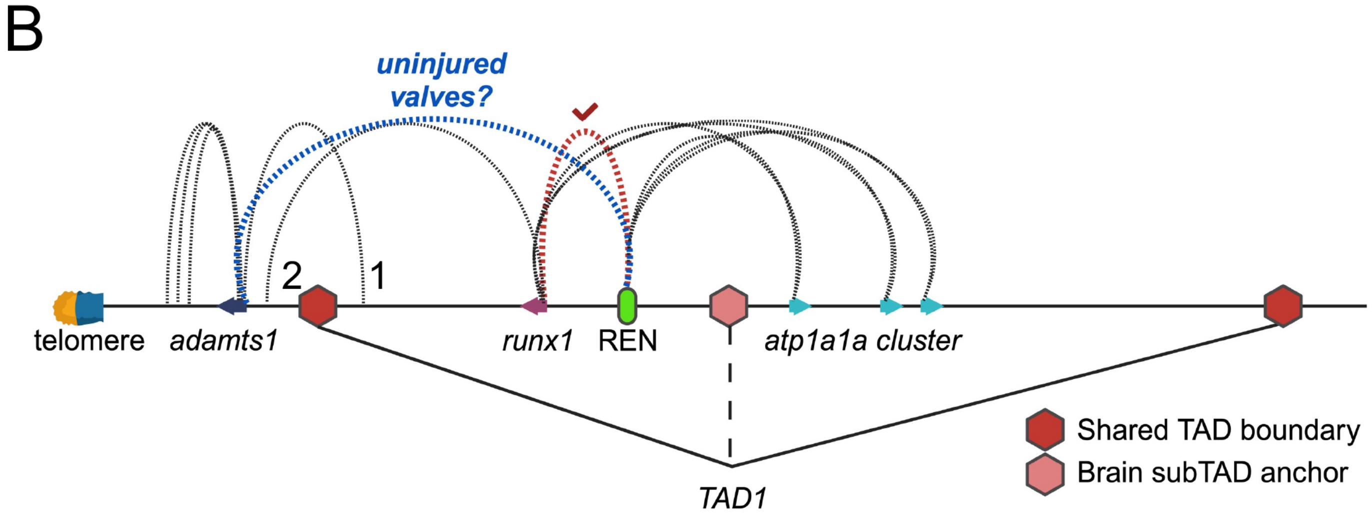

Fig. S6.

(A) The *REN* enhancer deletion effectively acts as a CM and epicardial knockout of *runx1* during regeneration. After injury *REN:GFP* only expresses in CMs and epicardium but *runx1* is also endocardial. Thus, knockout of *REN* has *wild type* endocardial expression of *runx1*. Proliferation phenotypes in Δ*REN* therefore arise from CMs or epicardium. (B) Cartoon of the entire region of chromosome 1 including the telomeric end and the TAD containing both *REN* and *runx1*. Genes are shown as arrows with dotted lines representing 3D interactions found by Hi-ChIP. The loop marked 1 is the interaction of the *adamts1* promoter with an enhancer within the TAD. The loop marked 2 is the interaction between the *runx1* and an enhancer outside of the TAD nearby *adamts1*. Created in BioRender by Goldman, A. (2025). https://BioRender.com/k15g754. This figure was sublicensed under CC-BY 4.0 terms.

Development • Supplementary information

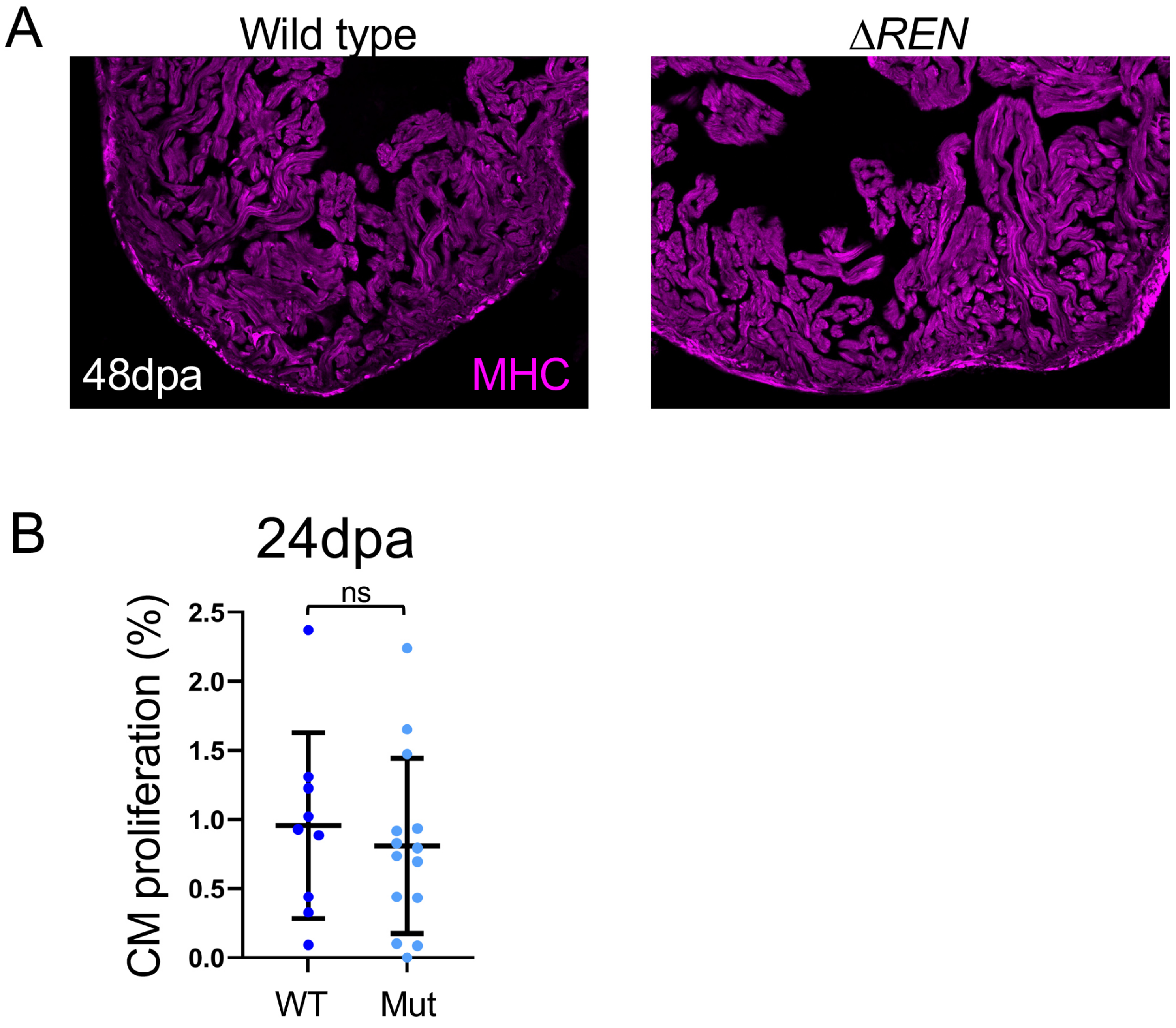

Fig. S7. The Δ*REN* mutants show no obvious phenotypes in muscle growth at the end of regeneration.

(A) No excess muscle growth was observed 48 days after amputation (dpa). Muscle was stained with an antibody directed towards the Myosin heavy chain (MHC). (B) There is no significant difference in CM proliferation indexes towards the end of regeneration (24 dpa).

Table S1. RNAseq analysis of Δ*REN* mutant hearts during regeneration. Lists of transcripts that significantly change in abundance.

Available for download at
https://journals.biologists.com/dev/article-lookup/doi/10.1242/dev.204458#supplementary-data

Table S2. RNAseq analysis of uninjured Δ*REN* mutant hearts. Lists of transcripts that significantly change in abundance.

Available for download at
https://journals.biologists.com/dev/article-lookup/doi/10.1242/dev.204458#supplementary-data

Table S3. Summary of Hi-C and tandem snRNA-seq/ATAC-seq data from Yang et al., 2020). All interactions with *REN*, *runx1,* and *adamts1* are collated.

Available for download at
https://journals.biologists.com/dev/article-lookup/doi/10.1242/dev.204458#supplementary-data
