# Supplementary material for: A cardiac transcriptional enhancer is repurposed during regeneration to activate an anti-proliferative program
Source: Development. 2025 Feb 17;152(4):DEV204458. doi: 10.1242/dev.204458 (PMC11883283; doi:10.1242/dev.204458)
Supplement: Supplementary file 1 [file develop-152-204458_review_history.pdf]

A cardiac transcriptional enhancer is repurposed during regeneration to activate an anti-proliferative program

Anupama Rao, Andrew Russell, Jose Segura-Bermudez, Charles Franz, Rejenae Dockery, Anton Blatnik, Jacob Panten, Mateo Zevallos, Carson McNulty, Maciej Pietrzak and Joseph Aaron Goldman

DOI: 10.1242/dev.204458

Editor: Benoit G. Bruneau

Review timeline

Original submission:	10 December 2023
Editorial decision:	18 January 2024
First revision received:	16 October 2024
Editorial decision:	19 November 2024
Second revision received:	6 December 2024
Accepted:	17 December 2024

Original submission

First decision letter

MS ID#: DEVELOP/2023/202576

MS TITLE: A cardiac transcriptional enhancer is repurposed during regeneration to activate an anti-proliferative program

AUTHORS: Anupama Rao; Andrew Russell; Jose Segura-Bermudez; Charles Franz; Anton Blatnik; Mateo Zevallos; Maciej Pietrzak and Joseph Aaron Goldman

I have now received all the referees' reports on the above manuscript, and have reached a decision. The referees' comments are appended below, or you can access them online: please go to BenchPress and click on the 'Manuscripts with Decisions' queue in the Author Area.

As you will see, the referees express considerable interest in your work, but have some significant criticisms and recommend a substantial revision of your manuscript before we can consider publication. If you are able to revise the manuscript along the lines suggested, which may involve further experiments, I will be happy to receive a revised version of the manuscript. Your revised paper will be re-reviewed by one or more of the original referees, and acceptance of your manuscript will depend on your addressing satisfactorily the reviewers' major concerns. Please also note that Development will normally permit only one round of major revision. If it would be helpful, you are welcome to contact us to discuss your revision in greater detail. Please send us a point-by-point response indicating your plans for addressing the referees' comments, and we will look over this and provide further guidance.

Please attend to all of the reviewers' comments and ensure that you clearly highlight all changes made in the revised manuscript. Please avoid using 'Tracked changes' in Word files as these are lost in PDF conversion. I should be grateful if you would also provide a point-by-point response detailing how you have dealt with the points raised by the reviewers in the 'Response to Reviewers' box. If you do not agree with any of their criticisms or suggestions please explain clearly why this is so.

© 2025. Published by The Company of Biologists under the terms of the Creative Commons Attribution License (https://creativecommons.org/licenses/by/4.0/).

Reviewer 1

Advance Summary and Potential Significance to Field:

The authors analyzed a runx1-linked enhancer (REN) previously identified from a H3.3 enrichment profile of regenerating zebrafish heart. Deletion of the genomic region encompassing REN (dREN) results in increased cardiomyocyte proliferation, similar to runx1 mutant phenotypes reported by Koth et al.
Interestingly, REN also drives gene expression in cardiomyocytes around valves and outflow tract of the heart, and its activity shifts from the cardiomyocytes around the valve to the regenerating area where REN activity peaks at 7 days post amputation during heart regeneration. Furthermore, increased collagen level in the valve is observed in dREN mutants.
The authors suggest that different genes are regulated by REN during heart regeneration and in the valve of uninjured hearts, and that REN regulates an anti-cardiomyocyte proliferation program.

Reviewer 1 Comments for the Author:

The manuscript is succinctly written but figure labels and nomenclature need to be checked and corrected. The imaging data are of high quality. Furthermore, the argument about the differences between their data and data from Koth et al is subject to different interpretations. Some data are missing and information is not provided to assess the results.

Major concerns:

1.It is unclear whether the quantification in Fig. 1G actually represents the difference between Fig. 1B and D. The GFP intensity in tcf21 negative cells (CM) is as strong if not stronger than tcf21 positive epicardium in Fig. 1D and 1D’. The images might be overly saturated for quantification of intensity. It is also unclear whether the authors are comparing 3dpa tcf21+ epicardium with 7 dpa MHC+ muscle instead of 3 dpa in Fig. 1G.
2.The genomic region (3162 bp) deleted using CRISPR encompassing REN is a lot bigger than REN (1265 bp). The authors should also show REN location in relation to this region in Fig. 3A. Is it possible that the surrounding sequences affect REN activity?
3.The authors did not provide RNAseq data of dREN mutant hearts as a supplemental table. fn1a is described in the text but not shown in Fig. 3E.
4.The authors should do RNAScope to check runx1 expression in dREN mutants and WT controls to characterize any cell-specific changes or help explain the data in Fig. 3G. Similarly, RNAScope should be performed to check adamts1 expression.
5.Is REN activity in myocardium or epicardium required for cardiomyocyte proliferation?
6.The scarring differences might be also affected by different injury models (amputation vs cryoinjury). The authors cannot rule out the possibility that the part of the deleted region in dREN mutant also affects/increases scar formation, which is counteracted by increased cardiomyocyte proliferation that is supposed to decrease scar formation.

The scar formation is hypothesized to be a balance or competition between cardiomyocytes and fibroblasts. In REN mutants with increased cardiomyocyte proliferation, collagen formation could be decreased. However, dREN mutants also have an independent function of increasing collagen. This could be the explanation that overall scar formation is not affected. The authors might consider discussing these aspects.

Minor comments:

1.The nomenclature of flk1 should be kdrl. –flk1:mCherry should be changed to kdrl:mCherry.
2.The tcf21:mCherry reporter should be tcf21:dsRed unless the tcf21:mCherry-NTR line was used. Based on the citation, it should be the tcf21:dsRed line.
3.Line 250, Fig. S4B should be Fig. S3B.
4.Line 274, Fig. S5C and Fig. S5D should be Fig. S3.
5.Line293—Fig. S4A should be Fig. 4G.
6.Line 305—"On occasion,” –needs to be delete as it is duplicated in the same sentence.

© 2025. Published by The Company of Biologists under the terms of the Creative Commons Attribution License (https://creativecommons.org/licenses/by/4.0/).

Reviewer 2

The manuscript authored by Rao et al. provides an insightful exploration into the functionality of the runx1-associated enhancer (REN) in the adult zebrafish heart. The study is a continuation of the corresponding author's previous research, which identified REN as a regulator of proliferating cells during zebrafish heart regeneration (Goldman et al., 2017). In this current investigation, the authors showed that REN controlled GFP expression around cardiac valves in uninjured hearts in addition to injury-induced expression in the epicardium and cardiomyocytes. Furthermore, the authors delineate specific regulatory elements within REN governing epicardial and cardiomyocyte expressions. The deletion of REN resulted in a reduction but not complete elimination of injury-induced runx1 upregulation, suggesting its essential yet not exclusive role as an enhancer during heart regeneration. Surprisingly, REN deletion mutant exhibited increased cardiomyocyte proliferation during regeneration, implying an anti-proliferative function of REN. Additionally, the authors observed a declined valve expression of the REN reporter during heart regeneration, which seemed to be unrelated to the runx1 activity. Through RNAseq and analysis of published Hi-C datasets, the authors predicted an association between the valve activity of REN and gene adamts1, which resides within the topologically associating domain of REN. The authors concluded they had revealed a new mechanism of gene regulation, in which an enhancer regulating valve-related genes is repurposed to activate a different gene in the regeneration site in zebrafish hearts. The manuscript is well-written, and the conclusion is of potential high interest in the field of gene regulation. However, the conclusion proposing a novel gene regulation mechanism appears somewhat premature and requires more substantial evidence for firm establishment.

Reviewer 2 Comments for the Author:
Major:
1. The authors claimed to have revealed a new mechanism of gene regulation. However, as noted in the manuscript, it is well-known that a single enhancer could interact with multiple promoters to regulate several genes. It would improve the study significantly with deeper elucidation of the underlying mechanisms (e.g., how REN was “repurposed”). It would also be helpful to be more specific in claiming the mechanistic advances.

2. Fig. 2: Expressions of short regulatory fragment-driven transgenes are commonly affected by the flanking genomic sequences. How many stable or founder lines have been tested to confirm the consensus expression patterns of each regulatory fragment?

3. Fig. 5: It was claimed that valves in the ΔREN mutant hearts had stronger collagen staining than the wildtype, which suggests a decreased abundance of cardiac valve associated Adamts1 that degrades collagen. However, this piece of data is not convincing without quantitative measures of collagen deposition.
In addition, the authors also failed to demonstrate the association of adamts1 mRNA and REN activity in the valve.

Minor:
4. Fig. 2A: It would be informative to label the 4 blocks on the cartoon.

5. Lines 274-277: Typo, the referenced figures should be Fig. S3, not S5.

6. Line 305, duplicate words “on occasion”.

First revision

Author response to reviewers' comments

SUMMARY – We thank the reviewers for the time and consideration that they put into this manuscript. The reviewers appreciated the question and approach of our study. However, the

© 2025. Published by The Company of Biologists under the terms of the Creative Commons Attribution License (https://creativecommons.org/licenses/by/4.0/).

reviewers asked for some further experiments to solidify some understanding. We have addressed each comment with either the experiments suggested or what we believe to be an equivalent. The manuscript is far better, and we thank the reviewers for their help.

Below we address each of the reviewer comments individually:

Reviewer 1 Advance Summary and Potential Significance to Field:
The authors analyzed a runx1-linked enhancer (REN) previously identified from a H3.3 enrichment profile of regenerating zebrafish heart. Deletion of the genomic region encompassing REN (dREN) results in increased cardiomyocyte proliferation, similar to runx1 mutant phenotypes reported by Koth et al. Interestingly, REN also drives gene expression in cardiomyocytes around valves and outflow tract of the heart, and its activity shifts from the cardiomyocytes around the valve to the regenerating area where REN activity peaks at 7 days post amputation during heart regeneration. Furthermore, increased collagen level in the valve is observed in dREN mutants. The authors suggest that different genes are regulated by REN during heart regeneration and in the valve of uninjured hearts, and that REN regulates an anti-cardiomyocyte proliferation program.

Reviewer 1 Comments for the Author:

The manuscript is succinctly written but figure labels and nomenclature need to be checked and corrected. The imaging data are of high quality. Furthermore, the argument about the differences between their data and data from Koth et al is subject to different interpretations. Some data are missing and information is not provided to assess the results.

We thank the Reviewer very much for the generous comments regarding our writing and data quality. The Reviewer's attention to detail is also appreciated.

Unfortunately, we have given the impression that our manuscript conflicts with Koth et al. and that is far from our position. We find the Koth et al. work to be of high-quality and rigor. However, using new reagents (□*REN*) we have learned new information that expands on what was reported. Koth et al. did not have the benefit of the □*REN* tool before making their conclusions. With the □*REN* tool, we conclude that *runx1* expression in either muscle or epicardium (or both) is critical for restricting CM proliferation.

However, we also acknowledge that the □*REN* tool cannot ***exclude*** a role for *runx1* in restricting CM proliferation from the endocardium as was suggested in Koth et al. manuscript. Rather, we can now only exclude endocardial *runx1* as the **sole** contributor to CM proliferation changes. We have modified our statements throughout the paper to make our position clearer and to be more rigorous. Our hope is that this alleviates the main concerns of Reviewer 1 and clarifies our appreciation of the work in Koth et al.

Please see revised manuscript:
Results
Lines 290-292: "These data suggest that the observed differences in scarring in *runx1* mutants are not the sole reason for observed increases in CM proliferation (please see Discussion for more details). *REN* controlled regulation by *runx1* in the epicardium and/or myocardium also effects CM proliferation."

Discussion
Lines 450-453: "We cannot exclude a possible role for endocardial *runx1* in regulating CM proliferation as well. However, the Δ*REN* mutant clearly establishes epicardial and/or muscle expression of runx1 as being critical for negatively regulating CM proliferation during regeneration. More work is needed to dissect the individual cell-type specific contributions and to identify direct targets of Runx1 that modify the ability to regenerate hearts."

Please see specific questions addressed below for more discussion.

1. **It is unclear whether the quantification in Fig. 1G actually represents the difference**

© 2025. Published by The Company of Biologists under the terms of the Creative Commons Attribution License (https://creativecommons.org/licenses/by/4.0/).

between Fig. 1B and D. The GFP intensity in tcf21 negative cells (CM) is as strong if not stronger than tcf21 positive epicardium in Fig. 1D and 1D'. The images might be overly saturated for quantification of intensity. It is also unclear whether the authors are comparing 3dpa tcf21+ epicardium with 7 dpa MHC+ muscle instead of 3 dpa in Fig. 1G.

The quantification in **Fig. 1G** demonstrates the differences between **Fig. 1A** and **Fig. 1C**. As Reviewer 1 pointed out, the *REN* signal from epicardium is saturated in the MHC in **Fig. 1B** to better visualize the muscle signal. The black and white signal in **Fig. 1B** and **Fig. 1D** simply represent the colocalization area (black) or not (green). These images are binary and do not convey intensity differences. When comparing intensity between muscle and epicardium, all signals were analyzed from within the same heart in three different channels under sub-saturating conditions (*REN*, MHC and *tcf21*).

2. **The genomic region (3162 bp) deleted using CRISPR encompassing REN is a lot bigger than REN (1265 bp). The authors should also show REN location in relation to this region in Fig. 3A. Is it possible that the surrounding sequences affect REN activity?**

Reviewer 1 is correct that our CRISPR deletion is larger than the fragment that we used in the reporter. This was out of necessity due to the highly repetitive transposon composition of the *REN* region. It is true that we cannot exclude that our larger deletion may include *cis*-regulatory elements unrelated to *REN*. However, there is zero evidence of other cardiac *cis*-regulatory elements in this region based on several chromatin profiling studies (Goldman et al., 2017, Cao et al., 2022, and Cordero et al. 2024). Also, the reporter data and the deletion mutant data are consistent with one another around the valves. We find it unlikely that our results are simply the result of a generous deletion. However, we now discuss this caveat in the manuscript as suggested by Reviewer 1.

Please see revised manuscript:
Results
Lines 173-176: "We note that the 3,162 deletion encompasses the entire 1265bp of the reporter region. There is no reported evidence of other *cis*-regulatory elements being located within the 3,162bp in adult zebrafish cardiac tissues (Cao et al., 2022; Cordero et al., 2024; Goldman et al., 2017)."

CONFIDENTIAL

Discussion
Lines 421-426: "We note that the 3,162bp deletion in the Δ*REN* mutant may remove a previously undetected *cis*-regulatory element that regulates valvular genes. However, the same minimal fragment of *REN* is necessary and sufficient to activate during regeneration is also necessary and sufficient to activate in uninjured valves (Fig. 4). Thus, the ▫*REN* deletion mutant and reporter experiments are consistent with one another suggesting the same enhancer element is driving expression in both contexts."

Methods
Lines 526-528: "The 3162bp deletion encompasses the entire 1265bp fragment from the reporter plus some adjacent sequence that consists of mostly transposons. Finding reliable sgRNA and genotyping primer pairs within highly repetitive regions was non-trivial."

3. **The authors did not provide RNAseq data of dREN mutant hearts as a supplemental table. fn1a is described in the text but not shown in Fig. 3E.**
We now provide three new Supplementary Tables :

Table 1 – ▫*REN* RNAseq analysis during regeneration with the calculations for fold change and linear regression.

Table 2 – Summary of the *REN*, *runx1* and *adamts1* contacts from Yue et al. 2020. Table 3 – ▫*REN* RNAseq analysis from uninjured hearts.

4. **The authors should do RNAScope to check runx1 expression in dREN mutants and WT controls to characterize any cell-specific changes or help explain the data in Fig. 3G. Similarly, RNAScope should be performed to check adamts1 expression.**

© 2025. Published by The Company of Biologists under the terms of the Creative Commons Attribution License (https://creativecommons.org/licenses/by/4.0/).

RNAscope has now been performed for *runx1* and *adamts1* as suggested by the reviewer. In Fig. 3FG we now show that *runx1* is reduced in CMs of ▫*REN* mutant hearts during regeneration. In Fig. 5AB we now show that *adamts1* is reduced in CMs that surround cardiac valves. These were wonderful suggestions and we thank the reviewer for improving the manuscript.

Please see revised manuscript:
Lines 264-278: "To determine the cell-type specific distribution of *runx1* expression we used RNAScope and a *runx1*-specific probe on wildtype and ▫*REN* mutant hearts (Fig. 3F). Co-staining of the *runx1* probe with an antibody towards the myosin heavy chain demonstrated that *runx1* mRNA decreases 65% in CMs of ▫*REN* mutant hearts (Fig. 3G, mean: wildtype = 183.3 and mutant = 82.13, p-value < 0.0001, N = 10 vs 8). We note that the remaining signal in the muscle may largely be background (see Material and Methods). We could not find a co-staining strategy that worked for epicardium or endocardium with RNAScope. However, non-CM signal from *runx1* mRNA also decreased 56% suggesting that *REN* controls expression in either epicardium or endocardium (Fig. 3G, mean: wildtype = 272.6 and mutant = 147, p-value = 0.0035, N = 10 vs 8). Due to the reporter expression pattern (see Fig. 1) we predict that it is in epicardial cells. The disappearing non-CM *runx1* mRNA occurs in cells invading into the wound in way similar to what is reported in the literature for epicardial cells (Fig. 3F, box 2 and 3 wildtype vs box 5 and 6 Δ*REN*)(Lepilina et al., 2006). In contrast, some of the remaining *runx1* mRNA is localized adjacent to cardiac muscle in regions reminiscent of endocardial cells (Fig. 3F, white triangles boxes 4, 5 and 6). We conclude that *REN* is a transcriptional enhancer of *runx1* expression in CMs and another cell-type(s) that is likely epicardium."
Lines 342-360: "However, another gene called *adamts1,* 672Mb away from *REN* did have decreased abundance (Table 1). The *adamts1* gene encodes an extracellular matrix protein that regulates cardiac valves by degrading collagen (Hong-Brown et al., 2015). We used RNAscope to determine changes in the spatial distribution of *adamts1* mRNA in wildtype and Δ*REN* mutant hearts. As expected, expression of *adamts1* mRNA was strong within endocardial interstitial cells that compromise cardiac valves (Fig. 5A, asterisks). It was also abundant in neighboring CMs (Fig. 5A, blue arrow) and likely epicardial cells (Fig. 5A, yellow arrow). In Δ*REN* mutant hearts, the abundance of *adamts1* transcripts decreased 62% in CM surrounding valves (Fig. 5B, mean: wildtype = 81.1and mutant = 31.1, Mann-Whitney p-value = 0.0001, N = 8 vs 9). Expression of *adamts1* also decreased to 66% in non-CM cells (Fig. 5C, mean: wildtype = 955.4 and mutant = 321.8, Mann-Whitney p-value = 0.0015, N = 9 vs 10). Therefore, we conclude that *REN* functions as a transcriptional enhancer for the *adamts1* gene in CMs and possibly in the epicardium surrounding cardiac valves. The expression of *adamts1* also increases at the site of injury during regeneration (Table 1). Interestingly, there was no significant difference between *adamts1* abundance in CMs at the site of injury between wildtype and Δ*REN* mutant hearts (Fig. 5C, mean: wildtype = 28.0 and mutant = 23.9, Welch's p-value = 0.634). The average level of *adamts1* mRNA is decreased 40% in epicardial cells although the p- value is not significant (Fig. 5C, mean: wildtype = 48.6 and mutant = 29.5, Welch's p-value = 0.088). We conclude that *REN* is not a critical enhancer for inducing *adamts1* in regenerating tissue but it's possible that *REN* fplays a more minor role as a shadow enhancer that stabilizes expression of *adamts1* in nonCMs."

5. **Is REN activity in myocardium or epicardium required for cardiomyocyte proliferation?**

   We do not know whether the effects on cardiomyocyte proliferation due to *REN* activity results from epicardial or myocardial *REN* regulation. Further evaluation would require new transgenic reagents and should be left for other studies since this is not the main point of the manuscript. However, in the studies shown here, we do establish one (or both) of these cell-types as important in mediating Runx1-dependent proliferation changes.

6. **The scarring differences might be also affected by different injury models (amputation vs cryoinjury).**

   We have done cryoinjury on ▫*REN* hearts and still see no differences in scar composition. We can include these data in the manuscript if Reviewer 1 insists but we choose to leave it as

© 2025. Published by The Company of Biologists under the terms of the Creative Commons Attribution License (https://creativecommons.org/licenses/by/4.0/).

data not shown.

The authors cannot rule out the possibility that the part of the deleted region in dREN mutant also affects/increases scar formation, which is counteracted by increased cardiomyocyte proliferation that is supposed to decrease scar formation.

Please forgive us but we could not follow the reasoning for this caveat.

The scar formation is hypothesized to be a balance or competition between cardiomyocytes and fibroblasts. In REN mutants with increased cardiomyocyte proliferation, collagen formation could be decreased. However, dREN mutants also have an independent function of increasing collagen. This could be the explanation that overall scar formation is not affected. The authors might consider discussing these aspects.

The regulation of Collagen in uninjured hearts appears to be based on *adamts1* and is certainly not based on *runx1*, whose mutants rescue the phenotype (see Fig. 5). Expression of *adamts1* increases during regeneration equally in wildtype and □*REN* mutants. There is no evidence for compensation by *adamts1*. Finally, we find the possibility that □*REN* is compensating for scar differences but not CM proliferation to be improbable. That conclusion would conflict with the Koth et al. manuscript, and we prefer not to propose that without having significantly more evidence.

Using the exact same techniques and methodology (a colorimetric assay) we could not find ‘scar differences’ as described by Koth et al. in our □*REN* mutants. Now we include a quantitative measure of Collagen using an antibody (please see Reviewer 2, Point 3). Expression of certain collagen genes does change dramatically in □*REN* mutant hearts during regeneration. We avoided highlighting this information initially because it requires additional investigation and addresses a question which isn’t the main point of this paper; that *REN* regulation represents a novel mechanism for gene regulation during regeneration (please see Reviewer 2, comment 1). Now we mention this data carefully in the Results section.

Please see revised manuscript:

Lines 292-296: “We note that the abundance of *collagen 12a1b*, which was previously reported to be pro-regenerative (Hu et al., 2022), increases only 11X during wildtype regeneration and 31X during regeneration of Δ*REN* mutant hearts (Table 1). Thus, it’s possible that *runx1* may also regulate CM proliferation in epicardium or muscle by changing the composition of the scar.”

CONFIDENTIAL

Minor comments.

1. **The nomenclature of flk1 should be kdrl. –flk1:mCherry should be changed to kdrl:mCherry.**
   We understand Reviewer 1’s preferred gene name and now use *kdrl*.

2. **The tcf21:mCherry reporter should be tcf21:dsRed unless the tcf21:mCherry- NTR line was used. Based on the citation, it should be the tcf21:dsRed line.**
   It is the tcf21:dsRed line and we have corrected all the text and images. Thank you very much for correcting that mistake before publication.

3. **Line 250, Fig. S4B should be Fig. S3B.**
   It has been corrected.

4. **Line 274, Fig. S5C and Fig. S5D should be Fig. S3.**
   Those have been corrected.

5. **Line293—Fig. S4A should be Fig. 4G.**
   That has also been corrected.

CONFIDENTIAL

6. **Line 305—"On occasion,” -needs to be delete as it is duplicated in the same sentence.**

© 2025. Published by The Company of Biologists under the terms of the Creative Commons Attribution License (https://creativecommons.org/licenses/by/4.0/).

Thank you. That sentence has been updated.

******* Reviewer 2 Advance Summary and Potential Significance to Field:**

Significance to Field:
The manuscript authored by Rao et al. provides an insightful exploration into the functionality of the runx1-associated enhancer (REN) in the adult zebrafish heart. The study is a continuation of the corresponding author's previous research, which identified REN as a regulator of proliferating cells during zebrafish heart regeneration (Goldman et al., 2017). In this current investigation, the authors showed that REN controlled GFP expression around cardiac valves in uninjured hearts in addition to injury-induced expression in the epicardium and cardiomyocytes. Furthermore, the authors delineate specific regulatory elements within REN governing epicardial and cardiomyocyte expressions. The deletion of REN resulted in a reduction but not complete elimination of injury-induced runx1 upregulation, suggesting its essential yet not exclusive role as an enhancer during heart regeneration. Surprisingly, REN deletion mutant exhibited increased cardiomyocyte proliferation during regeneration, implying an anti-proliferative function of REN. Additionally, the authors observed a declined valve expression of the REN reporter during heart regeneration, which seemed to be unrelated to the runx1 activity. Through RNAseq and analysis of published Hi-C datasets, the authors predicted an association between the valve activity of REN and gene adamts1, which resides within the topologically associating domain of REN. The authors concluded they had revealed a new mechanism of gene regulation, in which an enhancer regulating valve-related genes is repurposed to activate a different gene in the regeneration site in zebrafish hearts. The manuscript is well-written, and the conclusion is of potential high interest in the field of gene regulation. However, the conclusion proposing a novel gene regulation mechanism appears somewhat premature and requires more substantial evidence for firm establishment.

Reviewer 2 Comments for the Author:

Major Comments:

1. **The authors claimed to have revealed a new mechanism of gene regulation. However, as noted in the manuscript, it is well-known that a single enhancer could interact with multiple promoters to regulate several genes. It would improve the study significantly with deeper elucidation of the underlying mechanisms (e.g., how REN was“repurposed”). It would also be helpful to be more specific in claiming the mechanistic advances.**

   Previously, there have been two mechanisms reported for gene regulation during regeneration: 1) reactivation of embryonic programs (Kikuchi et al. 2010) and 2) invigoration of endogenous adult programs (Ogawa et al. 2021). Here we describe a third mechanism, where an enhancer is repurposed from a different cardiac domain and quite possibly even from a different gene. We have rewritten the introduction extensively to make this clearer.

   It is true, enhancer ‘hubs’ and promoter competition have been described previously where one enhancer co-regulates multiple genes. In the case of *REN*, it appears that single enhancer regulates two genes but not concurrently. Each is regulated in a different context and, interestingly, the expression between the two regions is inversely connected (see Fig. 4). This is a more incremental addition to the gene regulation field although very novel in the context of adult regeneration.

   We thank Reviewer 2 for forcing us to tighten up our reasoning and present a clearer analysis. The paper is much improved.

   Please see revised manuscript:
   Introduction
   Lines 66-74: “One critical question that is incompletely understood is where regeneration enhancers come from. In part, the regeneration genetic program involves reactivation of embryonic genetic elements, for example, promoter sequences for the cardiac transcription factor *gata4* (Kikuchi et al., 2010). Afterall, regeneration is largely a recapitulation of development (Goldman and Poss, 2020; Viragova et al., 2024). However, in the case of the heart, it is unclear how much of the developmental program is necessary. The transcription

CONFIDENTIAL

© 2025. Published by The Company of Biologists under the terms of the Creative Commons Attribution License (https://creativecommons.org/licenses/by/4.0/).

factor Klf1 is dispensable for embryogenesis but required for regeneration (Ogawa et al., 2021). Moreover, Klf1 expression alone is sufficient to cause massive cardiomyocyte hyperplasia by binding mostly to enhancers that are already present in the adult (Ogawa et al., 2021). However, the enhancer(s) most critical to Klf1-driven CM hyperplasia have yet to be identified."

Discussion
Lines 394-396: "In addition, here, we show that during regeneration, a particular enhancer is repurposed from functioning around cardiac valves and recruited to activate a different gene that is important for CM proliferation in regenerating tissue (Fig. 6A)."

2. **Fig. 2: Expressions of short regulatory fragment-driven transgenes are commonly affected by the flanking genomic sequences. How many stable or founder lines have been tested to confirm the consensus expression patterns of each regulatory fragment?**

   Reviewer 2 is correct. Insertional effects must be considered when evaluating transgenic reporters. For this reason, we select at minimum 2 and ideally 3 independent insertions to confirm expression patterns. For some of the fragments that were negative we selected up to 6 independent insertions. This was described in the methods section from a previous publication, and we include it again here. Thank you, Reviewer 2, for ensuring that we mention this very important and relevant detail.

   Please see revised manuscript:

   Lines 489-490: "For enhancer reporter lines, we selected at least 2 and as many as 6 independent insertions to exclude potential insertional effects."

3. **Fig. 5: It was claimed that valves in the ΔREN mutant hearts had stronger collagen staining than the wildtype, which suggests a decreased abundance of cardiac valve associated Adamts1 that degrades collagen. However, this piece of data is not convincing without quantitative measures of collagen deposition. In addition, the authors also failed to demonstrate the association of adamts1 mRNA and REN activity in the valve.**

   We now show quantitative changes in the abundance of Collagen I in ▫*REN* mutant valves using an antibody (see Fig. 5FG). We also show changes in the abundance on *adamts1* mRNA in *REN:GFP* expression domains around ▫*REN* mutant valves (see Reviewer 1 Major comment 4). These were wonderful suggestions that really strengthened the paper.

   Please see revised manuscript:

   Lines 373-382: "To quantitatively assess Collagen differences in Δ*REN* mutant valves we used immunofluorescence with an antibody towards Collagen I (Fig. 5F). In Δ*REN* mutant hearts, Collagen I increased 56% in valves (Fig. 5G, mean arbitrary density units: wildtype (blue) = 5691576603 and mutant (light blue) = 8904512333, Mann-Whitney p-value = 0.0091, N = 21 vs 20). This difference was rescued by crossing ▫*REN* to a ▫*runx1* mutant demonstrating that Collagen changes are independent of *runx1* (Fig. 5G, mean arbitrary density units: wildtype = 6545145907 and double het (purple) = 6671972330, Welch's p-value = 0.799, N = 13 vs 16). Taken together, deletion of *REN* results in both decreased abundance of a cardiac valve associated gene, *adamts1,* and in phenotypes associated with reduced Adamts1 function. We conclude that *REN* regulates *adamts1* expression around uninjured cardiac valves."

Minor:

4. **Lines 274-277: Typo, the referenced figures should be Fig. S3, not S5.**
   It has been corrected.

5. **Line 305, duplicate words "on occasion".**
   Thank you for finding that typo. It has been corrected.

CONFIDENTIAL
CONFIDENTIAL

© 2025. Published by The Company of Biologists under the terms of the Creative Commons Attribution License (https://creativecommons.org/licenses/by/4.0/).

Second decision letter

MS ID#: dev.204458

MS TITLE: A cardiac transcriptional enhancer is repurposed during regeneration to activate an anti-proliferative program

AUTHORS: Anupama Rao; Andrew Russell; Jose Segura-Bermudez; Charles Franz; Rejenae Dockery; Anton Blatnik; Jacob Panten; Mateo Zevallos; Carson McNulty; Maciej Pietrzak and Joseph Aaron Goldman

I have now received all the referees' reports on the above manuscript, and have reached a decision. The referees' comments are appended below, or you can access them online: please go to:

As you will see, the referees express considerable interest in your work, but have some significant criticisms and recommend a substantial revision of your manuscript before we can consider publication. If you are able to revise the manuscript along the lines suggested, which may involve further experiments, I will be happy to receive a revised version of the manuscript. Reviewer 4 indicated upon discussion that "the authors do not need to address the last point (testing REN activation in ZCAT induction) and potentially the third point, considering time constraints (physiological significance of REN mutations in valve function). Addressing the other points would be good to add mechanistic insight into the role of REN in regeneration and development." All other comments should be addressed. Your revised paper will be re-reviewed by one or more of the original referees, and acceptance of your manuscript will depend on your addressing satisfactorily the reviewers' major concerns. Please also note that Development will normally permit only one round of major revision. If it would be helpful, you are welcome to contact us to discuss your revision in greater detail. Please send us a point-by-point response indicating your plans for addressing the referees' comments, and we will look over this and provide further guidance.

Please attend to all of the reviewers' comments and ensure that you clearly highlight all changes made in the revised manuscript. Please avoid using 'Tracked changes' in Word files as these are lost in PDF conversion. I should be grateful if you would also provide a point-by-point response detailing how you have dealt with the points raised by the reviewers in the 'Response to Reviewers' box. If you do not agree with any of their criticisms or suggestions please explain clearly why this is so.

Reviewer 1: SUMMARY OF THE ADVANCE MADE IN THIS PAPER AND ITS POTENTIAL SIGNIFICANCE TO THE FIELD

SUGGESTIONS TO AUTHORS
The authors have addressed my major concerns. I have no further comments.

Reviewer 2: SUMMARY OF THE ADVANCE MADE IN THIS PAPER AND ITS POTENTIAL SIGNIFICANCE TO THE FIELD

The manuscript by Rao et al., has potential to be impactful for the understanding of zebrafish regeneration in the heart, by proposing a role for the runx1-enhancer (termed REN) in the adult zebrafish heart in uninjured vs injured/regenerative conditions.

SUGGESTIONS TO AUTHORS

Although the current data is extensive and the authors have been responsive to previous reviewer comments, I think that the data appears to insufficiently support the major conclusions that the enhancer is repurposed in the uninjured vs injured conditions.

To accomplish this, I would imagine that the authors would need to provide evidence that the REN region has chromatin contacts in the uninjured condition that changes in the injured/regenerative condition. Such an experiment could be challenging for many technical reasons.

© 2025. Published by The Company of Biologists under the terms of the Creative Commons Attribution License (https://creativecommons.org/licenses/by/4.0/).

Alternatively, one could show the potential for these contacts using relevant published datasets, which the authors have attempted. However, the meaning of the cartoon in Supp Fig 3F and the table is unclear to me. From the text, the implication is that REN and runx1 are within the same TAD from Hi-C data of adult zebrafish brain or skeletal muscle as shown in the cartoon and the table. Yet, the figure legend describes it as a cartoon for the complementation experiment. Despite the caveats of using this Hi-C data from other tissues of the zebrafish adult, I think that it would useful to add a panel that demonstrates the chromatin contacts between REN and runx1, and in the manuscript where appropriate, REN and adamts1, if these can be demonstrated.

The figure legend for the analogous cartoon in Supp Fig 6 refers to Hi-ChIP data that does not denote Yang et al. 2020, which should be referenced in the figure legend. As shown, the "uninjured valves?" in the cartoon conveys a model based on speculation in discussion rather than data that directly supports this model.

The observation that REN-driven expression in wildtype hearts in CMs surrounding the valves is "inversely correlated to expression at the site of injury" is interesting. In Figure 4, it would be helpful to the reader to outline the valve tissue, especially for panel 4B, similar to Figure 5, so that the reader can better assess the localization of the REN expression relative to the valve. An alternative conclusion could be that an upstream signal activates REN near the uninjured valve is also activated in the injured/regenerating heart. Although the authors mention the expression of the runx1-BAC construct in wildtype is not observed near valve tissue, what is the expression pattern by RNAscope of runx1 in the outflow tract in wildtype vs delREN? This would address spatial information that may not be apparent from the bulk RNAseq data for the various conditions.

In the abstract, the statement that "ΔREN Collagen phenotype is rescued even by Δrunx1 mutations" is not fully supported and should be modified. There is an apparent valve phenotype in the delREN homozygous mutant. Unless I am mistaken, the complementation experiment evaluated compound heterozygous mutants of delREN and runx1, not by testing if there is a change in the homozygous delREN phenotype by heterozygous loss of runx1.

On line 80, I think "track" should read "tract".

Reviewer 3

Reviewer 3: SUMMARY OF THE ADVANCE MADE IN THIS PAPER AND ITS POTENTIAL SIGNIFICANCE TO THE FIELD

In this manuscript Rao et al. have followed up on investigation of a previously described putative enhancer which is activated during cardiac regeneration, a 1265 bp fragment referred to as REN, located 103 kb upstream of the runx1 gene. Key unanswered questions are what gene(s) REN regulates and whether it is important for regeneration. They show that the REN:GFP (minimal promoter) transgenic reporter is activated in both muscle and epicardium following injury and made a limited series of transgenic reporter lines to try and map required sequences. The epicardial mapping was not conclusive, as some required sequences were mapped but sufficient sequences were not identified. Myocardial mapping was more convincing and indicated a region (including block 1, with an AP1 binding site) that is sufficient. Deletion of REN did not impair cardiac regeneration, and surprisingly even made it more efficient. Therefore, REN appears to function as anti-proliferative and gene expression changes caused by deltaREN include upregulation of several previously discovered pro-regenerative factors. ddPCR showed that runx1 is activated in deltaREN but only 57% of WT. It is not known if this is relevant to the phenotype. Also somewhat surprisingly, REN:GFP is also expressed in uninjured hearts, specifically in tissue around the OFT, and that this pattern goes away during regeneration. Many genes are also affected by deltaREN in the uninjured heart, including decreased levels of adamts1 in both CMs and non-CMs, and increased collagen staining is associated with deltaREN. Adamts1 is not in the same TAD as REN but is at least in cis and is a reasonable candidate for a REN target gene. The authors conclude that REN normally regulates adamts1 to modulate fibrosis around the OFT, and that during regeneration, it is used instead to regulate runx1 to limit proliferation.

© 2025. Published by The Company of Biologists under the terms of the Creative Commons Attribution License (https://creativecommons.org/licenses/by/4.0/).

The element mapping in the first part of the manuscript is not particularly informative and somewhat distracting from the main point of the study, but it is data relevant to REN and so no harm in publishing it here. The basic discovery of REN performing two distinct functions during homeostasis or regeneration is interesting, compelling, and novel. The study is somewhat frustrating, since of course it would have been much more straightforward if deltaREN impaired regeneration. But the results are clear and the observations will be of interest to those in the field. The manuscript has been revised once and appears improved. It is still not clear that runx1 is a key target during regeneration, or that adamts1 is a sufficient target of REN to modulate fibrosis in homeostasis. It would be good to investigate if runx1 and adamts1 hets show corresponding phenotypes, but it seems reasonable to suggest these as follow up experiments.

SUGGESTIONS TO AUTHORS

Major comment:

I have only one major comment, which is that the large disturbances in gene expression profiles caused by deletion of REN (a single putative enhancer) are surprising. Presumably these are mostly all downstream effects, but it might also just be noise. It would be useful to show the PC plots for the RNA-seq data sets comparing REN vs deltaREN in both homeostasis and during regeneration, just to be able to evaluate how different are these hearts.

Minor comments:

1) It would be useful to indicate the locations of the 4 CRM blocks on full length REN in Fig. 2A/B. They are shown in Supplemental data but most readers will not want to try and figure it out by going there. REN-deuce27 nomenclature is obscure.

2) The yellow text in Fig. 4G is impossible to read.

Reviewer 4

Reviewer 4: SUMMARY OF THE ADVANCE MADE IN THIS PAPER AND ITS POTENTIAL SIGNIFICANCE TO THE FIELD

In this work, Rao et al. reported differential functions of a genomic region within the zebrafish runx1 enhancer sequence in developmental and regeneration contexts. In their previous work (Goldman et al., 2017), they identified the genomic region called runx1-linked enhancer or REN, which is activated in proliferating cells within the wound of an injured heart. This current work identified shorter fragments within the 1265-bp REN element that become activated in cardiomyocytes and epicardial cells. Surprisingly, deletion of a 3,162-bp region that includes the REN element resulted in increased proliferation of CMs upon injury. This increased proliferation is linked to reduced runx1 expression in cardiomyocytes and epicardial cells, but not endocardial cells. Interestingly, REN mutations in non-injured hearts appear to affect mostly outflow valves through regulating a different gene, adamts1. There was a significant increase of Collagen I deposition in non-injured REN mutant valves, which were not associated with runx1 function.

The strengths of the paper include the establishment of the many transgenic lines to test the minimal sequence necessary to drive REN expression during regeneration and development as well as the generation of the REN mutants and the RNA-sequencing approach to find the possible genes regulated by this enhancer element. From a technical perspective, this manuscript is well done, and the experiments are well performed and interpreted. The proposed dual role of this enhancer element during development (in regulating adamts1) and regeneration (in regulating runx1) is also highly interesting and plausible.

A limitation of the paper is it does not provide a full mechanistic insight into the dual/differential role of REN in regeneration and development. I understand that there are significant technical/time challenges in testing epistatic interactions between REN and the candidate genes it regulates, for instance: in REN mutants, overexpressing runx1 during regeneration or adamts1

© 2025. Published by The Company of Biologists under the terms of the Creative Commons Attribution License (https://creativecommons.org/licenses/by/4.0/).

during development, and observe if the phenotypes were rescued; however, without these experiments, the functional link between REN and the regulated genes is not fully convincing.

SUGGESTIONS TO AUTHORS

Comments on the manuscript:
- What is the physiological consequence to the enhanced proliferation of CMs in the REN mutants? I.e. does the heart fully regenerate at an earlier timepoint (than the usual 90 days post injury), is there faster invasion of CMs into the injured zone?
- It is not clear to me the rationale behind the disappearance of REN:GFP expression in the myocardium around the outflow valve, which is its endogenous location in non-injured hearts - when the heart is injured until around 14 dpa. Mechanistically, can the authors discuss how these changes occur in cells that are much farther away from the injured ventricle apex?
- What is the physiological significance of the increased Collagen I deposition in REN mutant valves? Do the authors observe any valve tissue hardening, reduced blood flow due to thicker valves or retrograde blood flow?
- It is important to note that the role of adamts1 especially in zebrafish has not been elucidated; there have been no reports on mutant phenotypes and on functional link to outflow tract development. Similarly, in mice, Adamts1 mutants only exhibit a mild ventricular compaction phenotype. Can the authors discuss possibilities of other genes from the 158 downregulated genes that might also be regulated by REN in uninjured conditions, which might be involved in the Collagen overdeposition phenotype in the outflow valve?
- The authors proposed that REN functions by promoting runx1 expression during regeneration. Do the authors then observe even higher rates of cardiomyocyte proliferation or faster regeneration/scar resolving rates in double runx1; REN mutants or heterozygotes compared with REN mutants alone during regeneration?
- What is the expression of the CM-specific REN sequence in Figure 2E-H in response to ZCAT induction? The authors used the ZCAT system as a negative control, but why not also as a positive control - to see if the REN sequence is activated in any injury conditions, both amputation/physical and chemical?

Second revision

<u>Author response to reviewers' comments</u>

SUMMARY – We thank the reviewers for the time and consideration that they put into this manuscript. The reviewers appreciated the question and the quality of our study. They stated, “the basic discovery of *REN* performing two distinct functions during homeostasis or regeneration is interesting, compelling, and novel” **(Rev 3)** and that, “the proposed dual role of this enhancer element during development (in regulating *adamts1*) and regeneration (in regulating *runx1*) is also highly interesting and plausible” **(Rev 4)**. These two comments perfectly convey the basic premise of our manuscript and show that the Reviewers do not disagree.

The original reviewer **(Rev 1)** stated that they were satisfied with our resubmission. All the other Reviewers agreed that “current data is extensive, and the authors have been responsive to previous reviewer comments” **(Rev 2)**. With these comments and those mentioned above, it comes as a surprise that major revisions are still being requested.

Below we address each of the Reviewer comments individually:

Reviewer 1 Advance Summary and Potential Significance to Field:
The authors have addressed my major concerns. I have no further comments.

Reviewer 2 Advance Summary and Potential Significance to Field:
The manuscript by Rao et al., has potential to be impactful for the understanding of zebrafish regeneration in the heart, by proposing a role for the runx1-enhancer (termed REN) in the adult zebrafish heart in uninjured vs injured/regenerative conditions.

© 2025. Published by The Company of Biologists under the terms of the Creative Commons Attribution License (https://creativecommons.org/licenses/by/4.0/).

Major Comments:

1. ***Although the current data is extensive and the authors have been responsive to previous reviewer comments, I think that the data appears to insufficiently support the major conclusions that the enhancer is repurposed in the uninjured vs injured conditions. To accomplish this, I would imagine that the authors would need to provide evidence that the REN region has chromatin contacts in the uninjured condition that changes in the injured/regenerative condition. Such an experiment could be challenging for many technical reasons.***

   Using a CRISPR knockout we have entirely removed an enhancer from the genome. Then we observe that expression of *adamts1* is diminished only in uninjured hearts and *runx1* expression is only diminished during regeneration. Since we used RNAscope, we can see that these changes are occurring exactly where the enhancer dictates expression in a reporter. This data conclusively shows that *REN* regulates these two genes differently in the two different conditions. We have modified the first paragraph of the Discussion to make this clear.

   The limitation of these experiments is that they do not confirm whether regulation by *REN* is ***direct*** although this is clearly the simplest explanation. We used published Hi-C data sets to confirm the *REN-runx1* interaction but did not find contacts for *adamts1*. To address this, we provided the complementation experiment. While we cannot confirm that *REN* ***directly*** regulates *adamts1,* we have eliminated *runx1* as a possible intermediate. Thus, *REN* regulates two different and separable pathways in uninjured hearts and during regeneration.

   Whether *adamts1* is regulated directly or if there is a different intermediate does not change the major conclusion of the manuscript.

   Yes, it's true that determining the chromatin contacts would provide further confirmation. However, if contacts are not detected that does not invalidate our model. A complete understanding of how enhancers stimulate promoters is lacking and the importance of physical touch does not appear universal (Yang and Hansen, "Enhancer selectivity in space and time: from enhancer-promoter interactions to promoter activation", Nature Reviews Molecular Cell Biology, 25:574-591, 2024). Moreover, as the Reviewer pointed out, chromatin conformation experiments are technically challenging. Thus, a negative result from such experiments would be uninterpretable for the purposes of our study. We used the published literature instead (see below).

2. ***Alternatively, one could show the potential for these contacts using relevant published datasets, which the authors have attempted. However, the meaning of the cartoon in Supp Fig 3F and the table is unclear to me. From the text, the implication is that REN and runx1 are within the same TAD from Hi-C data of adult zebrafish brain or skeletal muscle as shown in the cartoon and the table. Yet, the figure legend describes it as a cartoon for the complementation experiment. Despite the caveats of using this Hi-C data from other tissues of the zebrafish adult, I think that it would useful to add a panel that demonstrates the chromatin contacts between REN and runx1, and in the manuscript where appropriate, REN and adamts1, if these can be demonstrated.***

   The figure legend for the analogous cartoon in Supp Fig 6 refers to Hi-ChIP data that does not denote Yang et al. 2020, which should be referenced in the figure legend. As shown, the "uninjured valves?" in the cartoon conveys a model based on speculation in discussion rather than data that directly supports this model.

   We agree with the Reviewer that leveraging published data sets is a reasonable way to establish whether regulation by *REN* is direct. In our submission we provided published chromatin contact data from Yang et al. that validates an interaction between *REN* and *runx1*. This suggests that *REN* regulation of *runx1* expression is likely to be direct. The panel that the Reviewer requested was provided as Fig. S3F and now we have clarified the figure legend and Table 3. For the *REN* and *adamts1* contacts, none were found in the tissues profiled in Yang et al. However, many contacts were found between *adamts1* and the TAD containing

© 2025. Published by The Company of Biologists under the terms of the Creative Commons Attribution License (https://creativecommons.org/licenses/by/4.0/).

REN-runx1. We highlight those data in Fig. S6B to demonstrate that the TAD boundary is flexible at the end of chromosome 1 and interactions between *adamts1* and *REN* are very feasible. We acknowledge in the Discussion that we cannot confirm that *REN* regulation of *adamts1* is direct. There is only one speculative interaction displayed in Fig. S6B (based on the data presented in our paper) and it was marked with a question mark. We can remove it if the Reviewer would like.

3. ***The observation that REN-driven expression in wildtype hearts in CMs surrounding the valves is "inversely correlated to expression at the site of injury" is interesting. In Figure 4, it would be helpful to the reader to outline the valve tissue, especially for panel 4B, similar to Figure 5, so that the reader can better assess the localization of the REN expression relative to the valve. An alternative conclusion could be that an upstream signal activates REN near the uninjured valve is also activated in the injured/regenerating heart.***

We do not provide any conclusion as to why or how *REN* expression is inversely correlated between the regions. The Reviewer is very likely correct that the same signal is activating *REN* in both locations. The temporal and quantitative dynamics of *REN* expression suggest that regulation of this signal may be coordinated at both ends of the heart. This is a fascinating phenomenon that we report here and that warrants further investigation in a follow up manuscript. We have now highlighted the *REN* expression regions in Fig. 4 as we had done in Fig. 5.

4. ***Although the authors mention the expression of the runx1-BAC construct in wildtype is not observed near valve tissue, what is the expression pattern by RNAscope of runx1 in the outflow tract in wildtype vs delREN? This would address spatial information that may not be apparent from the bulk RNAseq data for the various conditions.***

The expression of *runx1* is minimal around cardiac valves in uninjured hearts (see Figure). If detected, *runx1* is mainly confined to endocardial interstitial cells that compromise cardiac valves but unlike *adamts1* is nearly absent from *REN*-positive regions. This confirms that the *runx1*-BAC reporter is consistent with *in vivo* expression patterns of *runx1*. The *runx1*-BAC reporter contains the *REN* locus but does not express around valves suggesting that this activity of *REN* is unrelated to *runx1*. Please see lines 351-355 and Fig. 3H in the revised manuscript.
Interestingly, we used a *runx2b* probe as a positive control for the RNAscope and found that *runx2b* is highly expressed in and around valves and is likely the RUNX homolog that regulates normal valve development (**Fig. 1B**).

NOTE: We have removed unpublished data that had been provided for the referees in confidence.

***In vivo* expression of *runx1* mimics the *runx1*-BAC reporter. (A)** RNAscope show little expression of runx1 in REN -positive regions. **(B)** *runx2b* is expressed robustly in endocardial interstitial cells compromising the valve leaflets, and what appears to be epicardial and endocardial cells.

5. ***In the abstract, the statement that "ΔREN Collagen phenotype is rescued even by Δrunx1 mutations" is not fully supported and should be modified. There is an apparent valve phenotype in the delREN homozygous mutant. Unless I am mistaken, the complementation experiment evaluated compound heterozygous mutants of delREN and runx1, not by testing if there is a change in the homozygous delREN phenotype by heterozygous loss of runx1.***

We will change the sentence to say, "ΔREN Collagen phenotype is rescued even by **an allele with** Δrunx1 mutations".

Reviewer 3 Advance Summary and Potential Significance to Field:

In this manuscript Rao et al. have followed up on investigation of a previously described

© 2025. Published by The Company of Biologists under the terms of the Creative Commons Attribution License (https://creativecommons.org/licenses/by/4.0/).

putative enhancer which is activated during cardiac regeneration, a 1265 bp fragment referred to as REN, located 103 kb upstream of the runx1 gene. Key unanswered questions are what gene(s) REN regulates and whether it is important for regeneration. They show that the REN:GFP (minimal promoter) transgenic reporter is activated in both muscle and epicardium following injury and made a limited series of transgenic reporter lines to try and map required sequences. The epicardial mapping was not conclusive, as some required sequences were mapped but sufficient sequences were not identified. Myocardial mapping was more convincing and indicated a region (including block 1, with an AP1 binding site) that is sufficient. Deletion of REN did not impair cardiac regeneration, and surprisingly even made it more efficient. Therefore, REN appears to function as anti- proliferative and gene expression changes caused by deltaREN include upregulation of several previously discovered pro-regenerative factors. ddPCR showed that runx1 is activated in deltaREN but only 57% of WT. It is not known if this is relevant to the phenotype. Also somewhat surprisingly, REN:GFP is also expressed in uninjured hearts, specifically in tissue around the OFT, and that this pattern goes away during regeneration. Many genes are also affected by deltaREN in the uninjured heart, including decreased levels of adamts1 in both CMs and non-CMs, and increased collagen staining is associated with deltaREN. Adamts1 is not in the same TAD as REN but is at least in cis and is a reasonable candidate for a REN target gene. The authors conclude that REN normally regulates adamts1 to modulate fibrosis around the OFT, and that during regeneration, it is used instead to regulate runx1 to limit proliferation.

The element mapping in the first part of the manuscript is not particularly informative and somewhat distracting from the main point of the study, but it is data relevant to REN and so no harm in publishing it here. The basic discovery of REN performing two distinct functions during homeostasis or regeneration is interesting, compelling, and novel. The study is somewhat frustrating, since of course it would have been much more straightforward if deltaREN impaired regeneration. But the results are clear and the observations will be of interest to those in the field. The manuscript has been revised once and appears improved. It is still not clear that runx1 is a key target during regeneration, or that adamts1 is a sufficient target of REN to modulate fibrosis in homeostasis. It would be good to investigate if runx1 and adamts1 hets show corresponding phenotypes, but it seems reasonable to suggest these as follow up experiments.

Please see Major Comments 1 and 2 from Reviewer 2 where we explain how our manuscript conclusively shows that *REN* regulates *adamts1* only in the uninjured heart and *runx1* only during regeneration. We agree with the Reviewer that experiments with hets are more reasonable for a follow-up story.

Comments for the Author: Major Comments:

1. ***I have only one major comment, which is that the large disturbances in gene expression profiles caused by deletion of REN (a single putative enhancer) are surprising. Presumably these are mostly all downstream effects, but it might also just be noise. It would be useful to show the PC plots for the RNA-seq data sets comparing REN vs deltaREN in both homeostasis and during regeneration, just to be able to evaluate how different are these hearts.***

   We now provide the PCA plots (see Fig. S3E). In these plots you can see clear separation between WT and MUT samples suggesting significant gene expression differences between the two groups. As the Reviewer notes, very likely many of these changes are secondary effects and do not represent genes directly regulated by *REN*. It is worth considering that these gene expression differences captured in the adult encapsulate an entire lifetime of different biology since these mutants are not inducible. There may be additional unrecognized phenotypes at different stages beyond the two that we describe. Very likely heart biology is impacted enough to result in more secondary changes than would typically be expected.

   All the minor comments have been addressed. Thank you, the figures look much better.

Reviewer 4 Advance Summary and Potential Significance to Field:

© 2025. Published by The Company of Biologists under the terms of the Creative Commons Attribution License (https://creativecommons.org/licenses/by/4.0/).

In this work, Rao et al. reported differential functions of a genomic region within the zebrafish runx1 enhancer sequence in developmental and regeneration contexts. In their previous work (Goldman et al., 2017), they identified the genomic region called runx1-linked enhancer or REN, which is activated in proliferating cells within the wound of an injured heart. This current work identified shorter fragments within the 1265-bp REN element that become activated in cardiomyocytes and epicardial cells.

Surprisingly, deletion of a 3,162-bp region that includes the REN element resulted in increased proliferation of CMs upon injury. This increased proliferation is linked to reduced runx1 expression in cardiomyocytes and epicardial cells, but not endocardial cells. Interestingly, REN mutations in non-injured hearts appear to affect mostly outflow valves through regulating a different gene, adamts1. There was a significant increase of Collagen I deposition in non-injured REN mutant valves, which were not associated with runx1 function.

The strengths of the paper include the establishment of the many transgenic lines to test the minimal sequence necessary to drive REN expression during regeneration and development as well as the generation of the REN mutants and the RNA-sequencing approach to find the possible genes regulated by this enhancer element. From a technical perspective, this manuscript is well done, and the experiments are well performed and interpreted. The proposed dual role of this enhancer element during development (in regulating adamts1) and regeneration (in regulating runx1) is also highly interesting and plausible.

A limitation of the paper is it does not provide a full mechanistic insight into the dual/differential role of REN in regeneration and development. I understand that there are significant technical/time challenges in testing epistatic interactions between REN and the candidate genes it regulates, for instance: in REN mutants, overexpressing runx1 during regeneration or adamts1 during development, and observe if the phenotypes were rescued; however, without these experiments, the functional link between REN and the regulated genes is not fully convincing.

The main point of our manuscript is the “proposed dual role of this enhancer element during development (in regulating adamts1) and regeneration (in regulating runx1)”. The rescue experiments would validate ***which gene is leading to the observed phenotype***, rather than determine ***which gene the enhancer regulates***. The latter is the main point of the manuscript that we have clearly established using knockouts, ddPCR, and RNAscope. Also, since it is already published that *runx1* mutants have the same phenotype as the *REN* enhancer mutant, a rescue experiment like the one suggested strikes us as redundant (PMID: 32341028).

Moreover, the technical difficulties in doing these experiments cannot be overstated. The transgene MUST reflect endogenous expression levels because over-expression of Runx1 has been shown to function as a dominant negative (PMID: 28299651, PMID: 17684492, and PMID: 7530657) and over-expression of Adamts1 will likely also result in a phenotype. Thus, the transgenes themselves are very likely to create phenotypes of their own. We agree with the Reviewer that our main premise that enhancers change the program that they regulate during regeneration is “highly interesting and plausible”.

The Reviewer prefers more in-depth mechanistic analyses. While interesting, we believe that many of the requested experiments are tangential, and that we have sufficiently made the main point of the manuscript. No manuscript should be expected to solve every aspect of the model system. Requiring tangential experiments to publish serves to entrench larger powerful labs with abundant resources without significantly improving the quality of manuscripts. My lab is small, consisting of me, a technician, and a small cadre of undergraduates. Asking for a litany of experiments that do not address the main point from my lab would be devastating for our productivity and hamstrings our ability to get even minimal funding. In our humble opinion, we believe these requirements are hurting science more than they are helping. The community is better when even smaller labs can contribute something that is “interesting, compelling, and novel” (**Rev3**).

Reviewer 4 Comments for the Author:

© 2025. Published by The Company of Biologists under the terms of the Creative Commons Attribution License (https://creativecommons.org/licenses/by/4.0/).

1. ***What is the physiological consequence to the enhanced proliferation of CMs in the REN mutants? I.e. does the heart fully regenerate at an earlier timepoint (than the usual 90 days post injury), is there faster invasion of CMs into the injured zone?***

   We did not find regeneration to be completed earlier in our mutants (24dpa). However, we did not survey earlier time points since these experiments are labor and time intensive and do not speak to the main finding of our manuscript which concerns gene regulation by *REN* rather than the biology of *runx1*.

2. ***It is not clear to me the rationale behind the disappearance of REN:GFP expression in the myocardium around the outflow valve, which is its endogenous location in non-injured hearts - when the heart is injured until around 14 dpa. Mechanistically, can the authors discuss how these changes occur in cells that are much farther away from the injured ventricle apex?***

   We do not provide a rationale for this behavior without the data to back it up. Here, we report this phenomenon but look to future studies to unravel it. Reviewer 2 suggested that the same signaling pathway was responsible for *REN* activation in both regions. While that is very likely to be true, that does not explain how cross-organ coordination is possible.

3. ***What is the physiological significance of the increased Collagen I deposition in REN mutant valves? Do the authors observe any valve tissue hardening, reduced blood flow due to thicker valves or retrograde blood flow?***

   We do not provide any physiological significance to changes in valves. Those experiments would be very difficult to do in zebrafish and do not speak to the main point of our manuscript. If there is no physiological significance to the valves, that does not change the fact that *REN* directly or indirectly regulates *adamts1*. The Collagen phenotype is supportive of the RNAscope data showing down regulation of *adamts1* although other genes may also be involved (see below).

4. ***It is important to note that the role of adamts1 especially in zebrafish has not been elucidated; there have been no reports on mutant phenotypes and on functional link to outflow tract development. Similarly, in mice, Adamts1 mutants only exhibit a mild ventricular compaction phenotype. Can the authors discuss possibilities of other genes from the 158 downregulated genes that might also be regulated by REN in uninjured conditions, which might be involved in the Collagen overdeposition phenotype in the outflow valve?***

   We discuss the possibility of other genes being regulated by *REN* but do not see any obvious connections to valve biology (Please see lines 338-341). It is entirely possible, even likely, that *REN* regulates other genes besides *adamts1* (Please see Discussion lines 427-441) but the main point of our manuscript is that it in the uninjured heart what it regulates is separable from *runx1* (Figs. 5E and 5G).

   There are a pair of papers that link Adamts1 to cardiac valves in mice (PMID: 25615642 and PMID: 22180604). We now cite these two manuscripts. However, more research must be done to confirm these studies. Notably, the phenotypes reported in Fig. 1C of PMID: 25615642 reflect exactly what we observe in *REN* mutants. Regardless, many other Adamts factors have been reported to sculpt cardiac valves (PMID: 33352066). It is possible that some of these behaviors have been coopted by Adamts1 in zebrafish. It is also possible that the gene annotated as *adamts1* in fish is functionally equivalent to a different ADAMTS gene in mammals which routinely happens for large gene families.

5. ***The authors proposed that REN functions by promoting runx1 expression during regeneration. Do the authors then observe even higher rates of cardiomyocyte proliferation or faster regeneration/scar resolving rates in double runx1; REN mutants or heterozygotes compared with REN mutants alone during regeneration?***

© 2025. Published by The Company of Biologists under the terms of the Creative Commons Attribution License (https://creativecommons.org/licenses/by/4.0/).

We do not predict that the *runx1* mutation will be synergistic with the *REN* mutation. Both mutations lie in the same pathway and in either situation the expression of *runx1* is impaired. Whether cells lack Runx1 because it is not transcribed or because of a missense mutation this will not augment a phenotype resulting from the absence of Runx1.

6. ***What is the expression of the CM-specific REN sequence in Figure 2E-H in response to ZCAT induction? The authors used the ZCAT system as a negative control, but why not also as a positive control - to see if the REN sequence is activated in any injury conditions, both amputation/physical and chemical?***

   Full-length *REN* is activated during ZCAT induced injury (Please see Fig. 5D in PMID: 28245924). While we have not tested every minimal fragment of *REN* using ZCAT, we do not expect that their activity would be different then the full-length enhancer.

Third decision letter

MS ID#: dev.204458R1

MS TITLE: A cardiac transcriptional enhancer is repurposed during regeneration to activate an anti-proliferative program

AUTHORS: Anupama Rao; Andrew Russell; Jose Segura-Bermudez; Charles Franz; Rejenae Dockery; Anton Blatnik; Jacob Panten; Mateo Zevallos; Carson McNulty; Maciej Pietrzak and Joseph Aaron Goldman

I am happy to tell you that your manuscript has been accepted for publication in Development, pending our standard publication integrity checks.

Reviewer 1

Advance summary and potential significance to field

I have no further comments

Reviewer 3

Advance summary and potential significance to field

The authors provided the data requested, in terms of the PC plots. Indeed, the plots show significant mingling of the various samples, suggesting that the data is quite noisy and many of the differences unlikely to be biologically significant. This has no serious ramifications for the conclusions but I think its good to show the data in full transparency.

Comments for the author

No further issues.

Reviewer 4

Advance summary and potential significance to field

I defer to the editor, whose opinion and judgment I trust for the decision on this paper. I agree with Reviewer 2, who had mentioned that the evidence for repurposing of the enhancer is not necessarily supported by the data the authors had included in the manuscript.

© 2025. Published by The Company of Biologists under the terms of the Creative Commons Attribution License (https://creativecommons.org/licenses/by/4.0/).

I do want to discuss the response from the authors. When I was asked to review the paper, I was not aware that this had already gone through 1 round of revision, and as such, treated the manuscript as a new submission. This was an honest oversight on my part, and I acknowledge that I asked for too many experiments to strengthen the authors' conclusions.

That being said, I believe the authors' response to my comments are too personal, charged and accusatory, and do not address the scientific content of my comments. I understand that the authors are frustrated by the numbers of revisions, and sympathize with their reactions. My comments were written to improve the manuscript, strengthen the authors' data and ensure that the major conclusions are well supported. They do not have any relations to the size or reputation of the lab. Ultimately, science is a data-driven endeavour and the authors have to ensure to have the proper data (epistatic relationships to investigate upstream/downstream genetic interactions, functional data on heart/valve function) to draw the conclusions they did. I believe the authors would only benefit from refraining from using such personal language in rebuttal letters in the future.

© 2025. Published by The Company of Biologists under the terms of the Creative Commons Attribution License (https://creativecommons.org/licenses/by/4.0/).
